# Evolution of Ovipositor Length in *Drosophila suzukii* Is Driven by Enhanced Cell Size Expansion and Anisotropic Tissue Reorganization

**DOI:** 10.1016/j.cub.2019.05.020

**Published:** 2019-06-17

**Authors:** Jack E. Green, Matthieu Cavey, Emmanuelle Médina Caturegli, Benoit Aigouy, Nicolas Gompel, Benjamin Prud’homme

**Affiliations:** 1 Aix-Marseille Université, CNRS, IBDM, Institut de Biologie du Développement de Marseille, 13288 Marseille Cedex 9, France; 2 Ludwig-Maximilians Universität München, Fakultät für Biologie, Biozentrum, Grosshaderner Strasse 2, 82152 Planegg-Martinsried, Germany

**Keywords:** *Drosophila suzukii*, ovipositor, morphological scaling, tissue anisotropy, cell apical area

## Abstract

Morphological diversity is dominated by variation in body proportion [1], which can be described with scaling relationships and mathematical equations, following the pioneering work of D’Arcy Thompson [2] and Julian Huxley [3]. Yet, the cellular processes underlying divergence in size and shape of morphological traits between species remain largely unknown [4, 5, 6, 7, 8]. Here, we compare the ovipositors of two related species, *Drosophila melanogaster* and *D. suzukii*. *D. suzukii* has switched its egg-laying niche from rotting to ripe fruit [9]. Along with this shift, the *D. suzukii* ovipositor has undergone a significant change in size and shape [10]. Using an allometric approach, we find that, while adult ovipositor width has hardly changed between the species, *D. suzukii* ovipositor length is almost double that of *D. melanogaster*. We show that this difference mostly arises in a 6-h time window during pupal development. We observe that the developing ovipositors of the two species comprise an almost identical number of cells, with a similar profile of cell shapes and orientations. After cell division stops, we find that the ovipositor area continues to grow in both species through the isotropic expansion of cell apical area and the anisotropic cellular reorganization of the tissue. Remarkably, we find that the lengthening of the *D. suzukii* ovipositor compared to that of *D. melanogaster* results from the combination of the accelerated expansion of apical cell size and the enhanced anisotropic rearrangement of cells in the tissue. Therefore, the quantitative fine-tuning of morphogenetic processes can drive evolutionary changes in organ size and shape.

## Highlights

• *D. suzukii* has evolved a bigger egg-laying organ compared to *D. melanogaster*
• The inter-species size difference arises during a short development time window
• The fine-tuning of shared cellular processes drives this difference in morphogenesis
• The rate of cell apical area expansion and the tissue shape anisotropy have evolved

## Graphical Abstract

**Figure:**
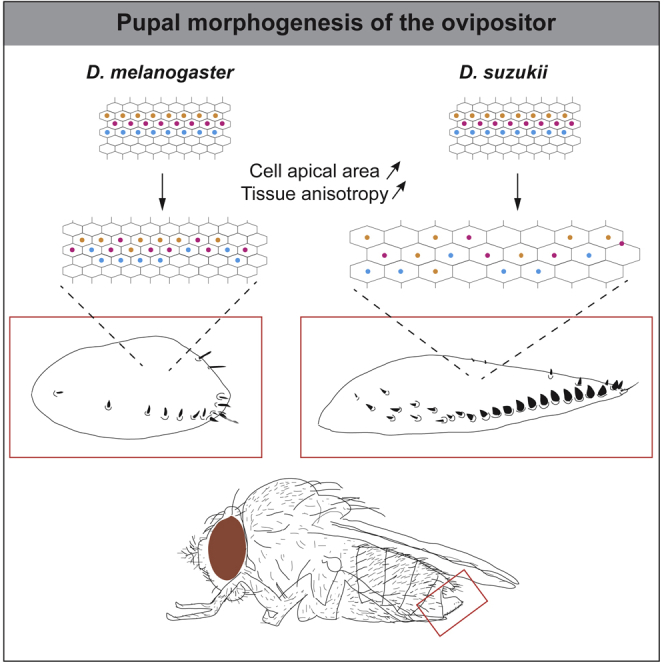

## Teaser

The fruit pest species *Drosophila suzukii* has evolved a bigger egg-laying organ compared to *D. melanogaster*. Green et al. show that the fine-tuning of shared cellular processes—namely, the rate of cell apical area expansion and the tissue shape anisotropy—drives the change in size and shape of the egg-laying organ of *D. suzukii*.

## Results and Discussion

### The *D. suzukii* Ovipositor Is Almost Twice as Long as That of *D. melanogaster*

**Figure 1:**
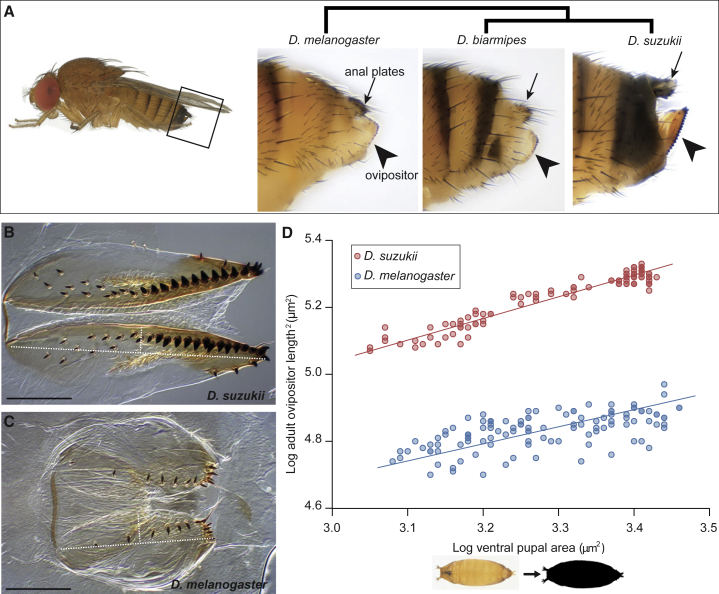
Evolutionary Shift in the Scaling Relationship of Ovipositor Length against Body Size between *D. suzukii* and *D. melanogaster* (A) Adult ovipositors of 3 closely related *Drosophila* species, *D. melanogaster*, *D. biarmipes*, and *D. suzukii*, in lateral profile (arrowhead indicates ovipositor, arrow indicates anal plates). The *D. suzukii* female is on the left; the boxed area indicates the approximate posterior region shown in the panels. Images are reproduced from [9]. (B and C) *D. suzukii* and *D. melanogaster* adult ovipositors, respectively. The long, white, dashed line indicates the measured length; the short indicates half width. The scale bar represents 200 μm. (D) Scaling relationship of ovipositor length squared and body size in *D. melanogaster* (blue; n = 114) and *D. suzukii* (red; n = 99) on a log-log scale. The overall body size is measured using ventral pupal area as a proxy, as illustrated on the x axis. The slope is modestly steeper in *D. suzukii*, increasing by 27% (*D. melanogaster* slope = 0.51, 95% CI = 0.44–0.59; *D. suzukii* slope = 0.65, 95% CI = 0.61–0.69; common slope test, p < 0.01). But more importantly, the intercept is shifted upward, indicating that ovipositor length is enlarged across the full range of body sizes. See also Data S1.

*D. suzukii* has evolved an enlarged ovipositor compared with those of its close relatives (Figure 1A). The dimensions of the ovipositor can be measured as a flattened plate (Figures 1B and 1C). There is a marginally significant, small difference in ovipositor width between the species (*D. melanogaster* width = 48 ± 1 μm; *D. suzukii* width = 52 ± 1 μm; Student’s t test, p = 0.02). Nevertheless, the major size difference is driven by the 1.6-fold increase in ovipositor length in *D. suzukii* (*D. melanogaster* length = 261 ± 2 μm; *D. suzukii* length = 414 ± 4 μm; Student’s t test, p < 0.001) (raw data are provided in Data S1). However, the length of the ovipositor most likely scales with overall body size, a phenomenon known as allometry [3, 11]. Since body size can vary between individuals and between species, we used an allometric approach to compare the scaling relationship between *D. melanogaster* and *D. suzukii*.

We generated a range of adult body size by systematically manipulating the diet of the flies. For both species, the log-transformed ovipositor squared length scales linearly with the log-transformed ventral pupal area, a proxy for body size (Figure 1D). It appears that, across all body sizes, the *D. suzukii* ovipositor is proportionally ∼60% longer than the *D. melanogaster* ovipositor. Therefore, the mechanism that determines the final ovipositor length for any given body size has diverged between species. We next sought to determine when the size difference in the ovipositor appears.

### The Interspecific Difference in Ovipositor Size and Shape Is Generated between 48 and 54 H in Pupal Development

The adult size in *Drosophila* is largely set by the larval growth [12]. Yet, to our surprise, we found that the difference in ovipositor size between *D. suzukii* and *D. melanogaster* has not appeared by the end of larval development (Figure S1). We therefore turned our attention to the ovipositor morphogenesis during pupal development.

**Figure 2:**
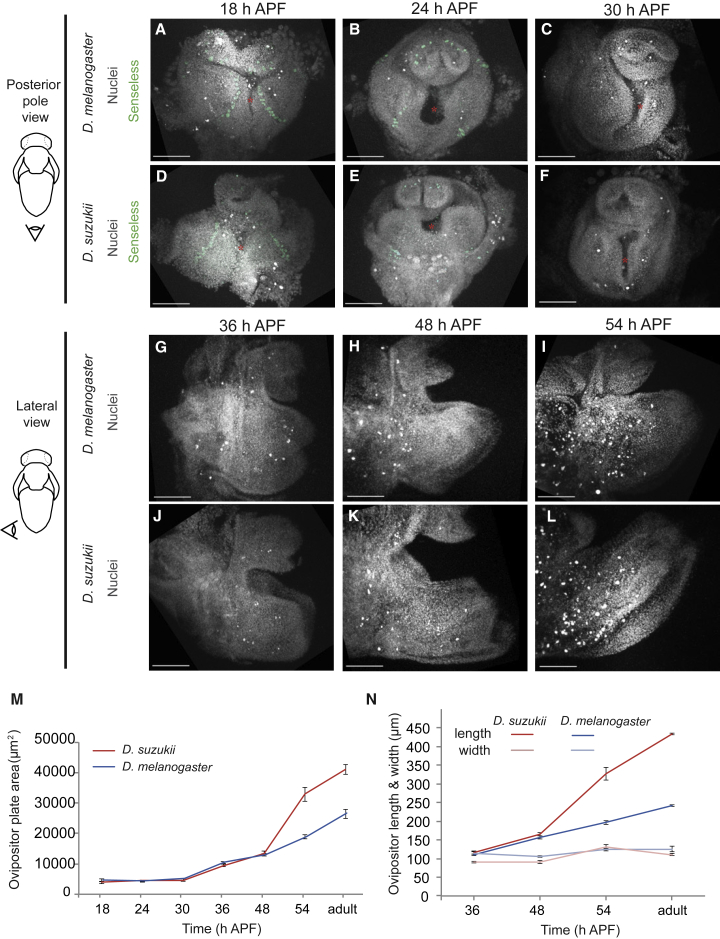
The Evolutionary Divergence in Ovipositor Size and Shape Is Generated in a Restricted Time Window during Pupal Development (A–F) Ovipositor development from 18 to 30 h APF in *D. melanogaster* (A–C) and *D. suzukii* (D–F), respectively. The presumptive ovipositor plates are arranged as a pair of lobes on either side of the future egg-laying cavity. (G–L) Ovipositor development from 36 to 54 h APF in *D. melanogaster* (G–I) and *D. suzukii* (J–L), respectively. The presumptive ovipositor projects out and elongates over this period. (A–L) All images are maximum projections of confocal stacks, with nuclei shown in gray and Senseless expression in green (A–F). A red asterisk indicates the egg-laying cavity. The schematic on the left illustrates the image orientation with respect to the pupa; images are posterior (A–F) or lateral (G–L) views. All images are to the same scale; the scale bar represents 50 μm. (M) Growth in the mean, total ovipositor plate area during metamorphosis in *D. melanogaster* (blue) and *D. suzukii* (red). n = 8–11 samples for each of the 7 time points. (N) Change in ovipositor plate length and width (bold and pale colors, respectively) over time in *D. melanogaster* (blue) and *D. suzukii* (red). n = 9 or 10 samples for each time point. In all graphs, error bars represent the standard error of the mean. See also Figures S1–S3; Data S1.

To address when the interspecific size difference first appears, we needed a set of markers to compare the ovipositor development between species. Between ∼18 and ∼30 h APF (hours after puparium formation), we found that in the presumptive ovipositor in both species, the gene *senseless* (*sens*) is expressed in a row of discrete cells, likely bristles precursor [13] (Figures 2A–2F). In parallel, the shape of the egg-laying cavity changes from a narrow triangular slit (∼18 h APF; Figures 2A and 2D) to a broader keyhole-like hollow (∼24 h APF; Figures 2B and 2E) and finally to a thinner, elongated cavity (∼30 h APF; Figures 2C and 2F). Soon after 30 h APF, the presumptive ovipositor starts to project out, shaping into a pair of plates, each made of two layers of cells (Figures 2G and 2J). The blades continue to elongate from 36 to 54 h APF in both species (Figures 2G–2L).

We measured the area of the presumptive ovipositor from 18 h APF to adulthood (Figure 2M) as well as its length and width when it starts to adopt the form of a pair of plates (Figure 2N). While ovipositor plate width modestly increases over development (by ∼10% in *D. melanogaster* and ∼20% in *D. suzukii*), ovipositor plate length increases substantially (by ∼140% in *D. melanogaster* and ∼260% in *D. suzukii*). Hence, within a given species, the increase in ovipositor plate area during development is chiefly due to an increase in length, thus transforming the shape of the tissue with time. Furthermore, most of the interspecific difference in ovipositor length is generated in a limited time window, between 48 and 54 h APF, and is subsequently maintained through later development and into the adult.

We therefore examined the cellular parameters that could explain the elongation of the ovipositor and its evolutionary divergence.

### Cell Proliferation Dynamics and Cell Numbers per Ovipositor Plate Are Very Similar between *D. melanogaster* and *D. suzukii*

We first asked whether the ovipositor size difference could be explained by divergence in cell proliferation. For all our cellular analyses, we examined exclusively the external cell layer of one plate per ovipositor, which is readily accessible for imaging after dissection. We first followed cell proliferation dynamics from 6 to 54 h APF using PH3 immunochemistry and found, in both species, that there is a final burst of cell division before 36 h APF (Figure S2A). This shows that cell division in the ovipositor plates has stopped at least ∼12 h before the interspecific size difference emerges. We can therefore exclude a contribution from cell division to the differential ovipositor elongation.

**Figure 3:**
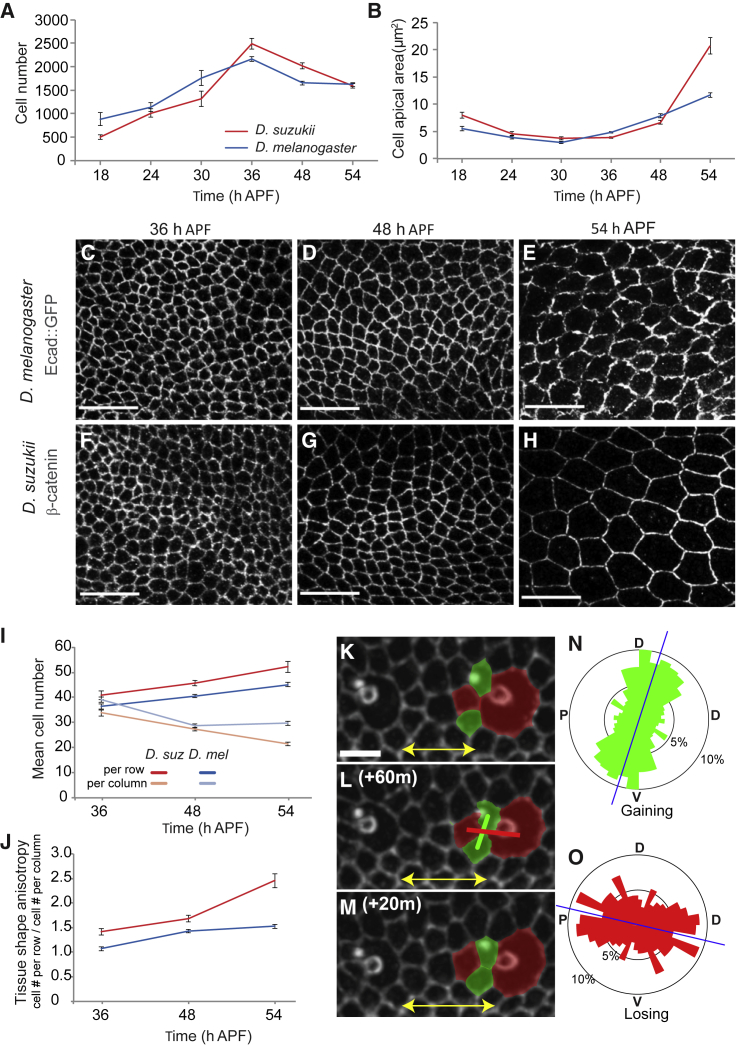
The Evolutionary Divergence in Ovipositor Size and Shape Is Driven by Accelerated Cell Size Expansion and Cell Reorganization (A and B) Change in mean, total ovipositor plate cell number (A) and in mean cell apical area (B) during pupal development in *D. melanogaster* (blue) and *D. suzukii* (red). n = 8–13 for each time point. (C–H) Illustrative examples of cells in the developing ovipositors of *D. melanogaster* (C–E) and *D. suzukii* (F–H) at 3 time points during ovipositor elongation. *D. melanogaster* Ecad::GFP was stained for GFP, and *D. suzukii* wild type was stained for β-catenin to reveal cell apical membranes, shown in gray. All images are to the same scale; the scale bar represents 10 μm. (I) Change in the mean number of cells per row and per column (bold and pale colors, respectively) in *D. melanogaster* (blue) and *D. suzukii* (red). (J) Changes in tissue shape anisotropy (cell number per rows divided by cell number per columns) in *D. suzukii* and *D. melanogaster*. n = 9–10 for each time point. In all graphs, error bars represent the standard error of the mean. (K–M) Time lapse showing T1 transitions occurring between two sensory bristles (large round cells). Green cells gain contact (convergence); red cells lose contact (divergence). Thick green and red bars indicate the axes of convergence and divergence, respectively. Yellow double-headed arrows mark the increasing distance between bristles. The scale bar represents 5μm. (N and O) Angular distributions of cell convergence axes (N) and cell divergence axes (O). Blue bars, average angles with respect to the PD axis (as described in [15]). Stable T1 transitions (n = 391, from 3 individuals) were observed during 8 to 12 h starting at ∼36 h APF. See also Figures S2 and S3; Data S1; and Videos S1 and S2.

To investigate further the connection between cell behavior and ovipositor growth, we used a transgenic *D. melanogaster* line containing DE-cadherin fused to GFP [14] and anti-β-catenin antibody staining in *D. suzukii* to label apical cell membranes. These markers for adherens junctions allow us to segment almost every cell across the external layer of an entire ovipositor plate (Figure S3), facilitating the extraction of quantitative cell parameters. Using this dataset, we found that the pattern of change in ovipositor plate cell number over time is almost identical in the two species (Figure 3A). Moreover, in both species, the total cell number actually declines by ∼30% from 36 to 54 h APF. Ultimately, we found no significant difference in cell number at 54 h APF (*D. melanogaster* = 1,619 ± 45 cells; *D. suzukii* = 1,594 ± 51 cells; Student’s t test, p > 0.05). We conclude that differences in total cell number per ovipositor plate cannot explain the difference in ovipositor length.

### The Evolutionary Difference in Ovipositor Size Is Driven by Accelerated Expansion of Cell Apical Area

We next examined if changes in mean cell apical area could account for the ovipositor size difference (Figure 3B). We found that initially, from 18 to 30 h APF, cell size roughly halves in both species. This period coincides with the final wave of cell division in the tissue (Figure S2A), and it is likely that these divisions are responsible for the cell size decrease [15]. However, from 36 h APF, after the cessation of cell division, apical cell surface starts to increase, in parallel with the ovipositor area (Figures S2B and S2C). From 36 to 48 h APF, cell apical area expands by ∼70% in both species (Figures 3B–3D, 3F, and 3G). In stark contrast, from 48 to 54 h APF, while cells area continues to expand in *D. melanogaster* by ∼50%, it expands by more than 200% in *D. suzukii*. By 54 h APF, an almost 2-fold difference in cell apical area has been generated that underpins the interspecific difference in ovipositor size (Figures 3B, 3E, and 3H). Importantly, we found no significant difference in mean cell size in pupal wings at 54 h APF between the species (Figure S2D). Hence, the more pronounced apical expansion of cells in the ovipositor plate is not a general feature of *D. suzukii* development but rather seems to be specific to its ovipositor. A common mechanism for cell size expansion is polyploidy [16], which can be measured using nuclear size [17]. However, in both species, we found no increase in nuclear area from 48 to 54 h APF (Figure S2E), arguing that the cell size expansion is not driven by polyploidy. Besides, we noted that cell surface area also expands in the *D. melanogaster* pupal wing, starting at roughly the same time point (∼32 h APF) [18], presumably by cell flattening [19]. Therefore, based on similarities between the pupal wing and the ovipositor epithelia, we assume that cell apical area expansion in the ovipositor plate results in epithelium flattening.

### The Global Patterns of Cell Shape and Orientation Are Very Similar between Species

To reconcile the uniform apical expansion of the cells and the elongation of the ovipositor, we examined two further cellular processes: cell elongation and cell orientation. We wondered whether cell size apical expansion was isotropic, thus preserving cell elongation, or whether it was anisotropic, thus stretching the cells. We measured cell elongation by fitting an ellipse to each segmented cell and calculating the ratio of the long and short axes of the fitted ellipse. To our surprise, the considerable expansion in cell apical area occurs essentially in an isotropic manner in both species. We found only minor and transient increases in cell elongation during the period of expansion, in particular in *D. suzukii* at 48 h APF (Figure S2F). Similarly, we found that, in both species, a similar proportion of cells (∼50%–55%) have their long axis aligned with the proximo-distal (PD) axis of the ovipositor at 54 h APF (Figure S2G). As for cell shape, we found at 48 h APF a transient increase in this proportion, specifically in *D. suzukii.* These observations suggest that at 48 h APF, the ovipositor is momentarily undergoing a stronger deformation in *D. suzukii* than in *D. melanogaster*. Therefore, although the exaggerated, isotropic cell size expansion in *D. suzukii* accounts for a fraction of the difference in ovipositor size between the species, changes in the global pattern of cell elongation or orientation cannot explain either the ovipositor elongation during development or the interspecific difference in shape.

### The Evolutionary Difference in Ovipositor Shape Is Driven by Anisotropic Reorganization of the Tissue

Next, we considered the ovipositor anisotropy by examining the organization of the cells in the external layer of the plate along the two major axes of the ovipositor—namely, the long, PD axis of the ovipositor (made of cell rows) and the orthogonal, short dorso-ventral (DV) axis (made of cell columns) (Figure S2H). We calculated the average number of cells per row and per column for each time point (Figure 3I), and we determined tissue shape anisotropy by finding the ratio between these mean numbers (Figure 3J).

In both species, the mean number of cells per row increases by 25% from 36 to 54 h APF (Figure 3I; *D. melanogaster =* 36 ± 1 to 45 ± 1 cells; *D. suzukii* = 41 ± 2 to 52 ± 2 cells; Student’s t test, p < 0.01). This suggests that, within each species, the increase in cell number along the PD axis contributes to the ovipositor elongation during development. Regarding the between-species difference, from 36 h APF, there is a consistent, small, but significant difference in the mean number of cells per row between *D. melanogaster* and *D. suzukii* (at 54 h APF, *D. melanogaster* = 45 ± 1 cells; *D. suzukii* = 52 ± 2 cells; Student’s t test, p < 0.05). Therefore, this ∼15% difference in mean cell number per row makes a further contribution to the interspecific difference in ovipositor length.

In contrast to ovipositor length, ovipositor width remains largely constant throughout development in both species (see Figure 2N). Interestingly, from 36 to 54 h APF, the mean number of cells per column declines in both species—by ∼24% in *D. melanogaster* and by ∼37% in *D. suzukii* (Figure 3I). This observation makes sense of the changes in ovipositor shape over time (Figure 2N). It shows that even though the cell expansion is isotropic, there is a compensatory reduction in the number of cells along the width of the ovipositor. Overall, uniform cell expansion is opposed by the reduction in column cell number, thus generating modest changes in width while length is substantially increased. These local changes in the tissue organization increase its global anisotropy—and therefore its length—during development both in *D. suzukii* in *D. melanogaster*. However, the anisotropy increase is stronger in *D. suzukii* than in *D. melanogaster* (Figure 3J), resulting in a longer ovipositor.

This reorganization of the cells in the tissue is likely to result from cell intercalations. To test this idea, we used live imaging in *D. melanogaster* to precisely assess cell intercalation and its contribution to the ovipositor plate remodeling from 36 h APF onward (Video S1). We observed cell intercalation in all directions, but a majority of the cells are gaining contacts along the PD axis, while a majority of cells are losing contacts along the DV axis (Figures 3K–3O). This means that, overall, cell rearrangements increase the number of cells along the PD axis while diminishing the number of cells along the DV axis. Therefore, cell intercalations contribute to the elongation of the ovipositor plate in *D. melanogaster*.

Video S1. T1 Transitions in *D. melanogaster* Ovipositor between 36 and 44 H APF, Related to Figure 3 115 stable T1 transitions were tracked in a clone of 233 cells (light yellow). Cells gaining, losing or gaining and simultaneously losing a contact are colored in green, red and yellow, respectively. The cell intercalation shown in Figure 2K is extracted from this movie. Proximal, left; Dorsal, top. Scale bar = 5μm

In addition, within each species, we noticed a striking agreement in the magnitude and timing of the changes in total cell number and in mean cell number per column per ovipositor plate (Figures S2I and S2J). This suggests that spatially patterned apoptosis or cell extrusion might contribute to the ovipositor elongation. However, our live imaging of *D. melanogaster* ovipositor did not reveal extensive or patterned cell elimination (Video S2). One limitation is that we have only been able to examine the external face of the ovipositor plate. Hence, we cannot rule out that, as a result of rearrangements or movements, some cells, rather than dying, roll around the contour of the plate and so move beyond our field of view.

Video S2. *D. melanogaster* Ovipositor Morphogenesis from 36 to 59 H APF, Related to Figure 3 The movie shows the external layer of one ovipositor plate (the other one is out of view). The sensory precursor cells are recognizable by their size and shape at the margin of the plate. The elongation of the ovipositor parallels its traction toward the abdomen (to the left). The pupal cuticle (visible at the bottom and then on the right) follows this movement toward the abdomen. Movies from two individuals were concatenated to cover the full period of interest (hence the slightly different orientations of the ovipositor in the two parts of the movie). Proximal, left; Dorsal, top. Scale bar = 5μm

### Differences in Cell Size and Tissue Shape Anisotropy Are Quantitatively Sufficient to Explain the Evolutionary Divergence in Ovipositor Length

**Figure 4:**
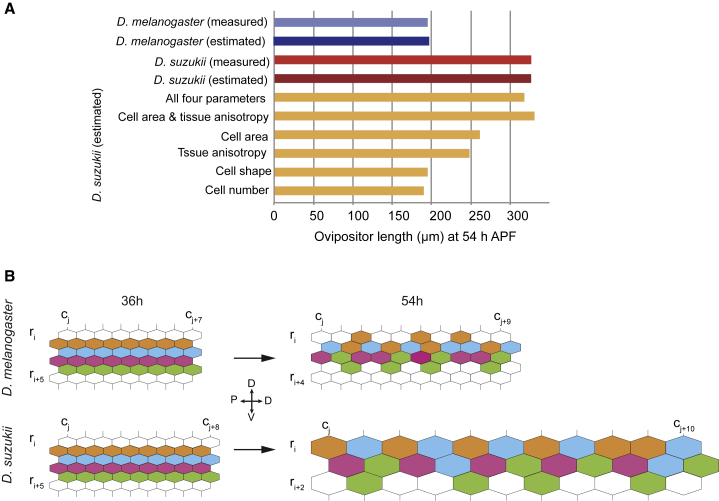
Modeling the Development and Evolution of Ovipositor Length Shows That the Measured Differences in Cell Size and Tissue Anisotropy Are Sufficient to Explain the Bulk of the Evolutionary Divergence (A) Different model estimates for ovipositor length compared with the measured value for ovipositor length in *D. melanogaster* (blue bars) or *D. suzukii* (red bars) at 54 h APF. The *D. melanogaster* (dark blue bar) or *D. suzukii* (dark red bar) estimates are calculated using the species-specific measured cellular parameters. To assess the relative contribution of the different cellular parameters to the evolutionary divergence, the *D. suzukii* length estimates (yellow bars) are calculated using the *D. melanogaster* values corrected with transformation coefficients for either (1) all four parameters, (2) cell area and tissue anisotropy together, or (3) each cellular parameter in isolation. (B) Schematic representation of the cellular changes that drive ovipositor elongation, in *D. melanogaster* (upper panel) and *D. suzukii* (lower panel), during pupal development from 36 to 54 h APF. Cells are idealized as hexagons and the ovipositor tissue as a hexagonal tessellation, oriented with the proximo-distal (PD) axis running left-to-right and the dorso-ventral (DV) axis running top-to-bottom. Rows and columns are arbitrarily labeled to reflect the change in their number during development and their difference between species. The schematic illustrates the increase in tissue anisotropy through the increase in the number of cells per row and the concomitant reduction in the number of cells per column during elongation, which acts to balance the isotropic expansion in cell area, thus reducing the net growth in ovipositor width. Colored cells mark neighboring cells in a particular row at 36 h APF. By 54 h APF, a substantial fraction of the cells have intercalated with one another, contributing further to the tissue elongation.

Next, we wanted to quantitatively describe how the different cellular parameters we identified could account for the measured ovipositor length differences. We used a simple model for the ovipositor and derived a mathematical equation to calculate the average ovipositor length based on the average total number of cells, tissue anisotropy, cell apical area, and cell shape (see STAR Methods). Focusing on the 54 h APF time point, we compared the model length estimates with the measured values for each species (Figure 4A). We then introduced coefficients into our mathematical equation to model the extent to which the measured between-species differences in cell parameters are sufficient to transform the *D. melanogaster* into the *D. suzukii* ovipositor and to assess their respective contributions to length divergence.

We found that the estimated ovipositor lengths at 54 h APF are in very good agreement with the measured values for both *D. melanogaster* and *D. suzukii* (Figure 4A). Furthermore, applying the transformation coefficient to the *D. melanogaster* parameter values yielded an estimated length that closely matches the value measured in *D. suzukii* (the difference is only 2.6%) (Figure 4A). This agreement suggests that our mathematical equation contains all the relevant parameters, and their respective quantitative changes, that are sufficient to estimate correctly the ovipositor lengths and to account for its transformation between *D. melanogaster* and *D. suzukii*. Furthermore, our numerical analysis shows that, together, changes in cell apical area and tissue anisotropy can be sufficient to account for the observed between-species ovipositor elongation (the difference between the estimated and the measured length is only 1.5%) (Figure 4A).

In conclusion, the quantitative changes in two shared cellular processes—namely, the rate of expansion of cell apical area and the rearrangements of the cells in the tissue that set the tissue’s anisotropy—could explain most of the difference in ovipositor length between *D. melanogaster* and *D. suzukii* (Figure 4B). We note, however, that we do not know whether the ovipositor cells are autonomously driving their expansion and reorganization [20] or instead are passively responding to some external forces applied to the tissue or a combination of both, as seen in other systems [15, 21, 22].

Consistent with the notion of a global force shaping the ovipositor development, we observed in our *D. melanogaster* movies that the ovipositor is pulled toward the abdomen, perhaps by cell contractions at the base of the ovipositor, as in the pupal wing [15]. This pulling oriented along the PD axis could create the force driving the oriented cell intercalations along the PD axis. In turn, the difference between *D. suzukii* and *D. melanogaster* would result from divergence in pulling intensities and therefore differences in the external forces exerted on the ovipositors. The transient elongation of the cells and the increase in the proportion of cells oriented along the PD axis, specifically in *D. suzukii*, at 48 h APF, just before the accelerated expansion of cell apical area, are consistent with this idea. Mechanical perturbations of ovipositor morphogenesis in *D. suzukii* will allow testing this hypothesis in the future.

## STAR★Methods

### Key Resources Table

**Table:** 

REAGENT or RESOURCE	SOURCE	IDENTIFIER
**Antibodies**		

anti-phospho-Histone H3 Ser10	Millipore	Cat# 06-570; RRID:AB_310177
Anti-Ubx/Abd-A (mouse)	DSHB	CAT#FP6.87; RRID:AB_10660834
Anti-E-Cadherin (rat)	DSHB	CAT#DCAD2; RRID:AB_528120
anti-armadillo	DSHB	CAT#N2 7A1; RRID:AB_528089
anti-Discs large	DSHB	CAT#4F3; RRID:AB_528203
anti-Senseless (guinea pig)	H. Bellen	N/A
Anti_Tsh (guinea pig)	R. Voutev; R. Mann	N/A
Anti-RFP (rabbit)	Rockland	Cat# 600-401-379S; RRID:AB_11182807
anti-GFP (rabbit)	ThermoFisher Scientific	Cat# PA1-29749; RRID:AB_1958064
anti-guinea pig AlexaFluor 488 (donkey)	Jackson ImmunoResearch	Cat# 106-545-003; RRID:AB_2337438
anti-mouse AlexaFluor 488 (donkey)	ThermoFisher Scientific	Cat# A-21202; RRID:AB_141607
anti-mouse AlexaFluor 568 (donkey)	ThermoFisher Scientific	CAT#A10037; RRID:AB_2534013
anti-mouse AlexaFluor 647 (donkey)	ThermoFisher Scientific	CAT#A-31571; RRID:AB_162542
anti-rat AlexaFluor 488 (goat)	ThermoFisher Scientific	CAT#A-11006; RRID:AB_2534074
anti-rabbit AlexaFluor 488 (goat)	ThermoFisher Scientific	CAT#A-11008; RRID:AB_143165
anti-rabbit AlexaFluor 568 (goat)	ThermoFisher Scientific	CAT# A-11011; RRID:AB_143157
anti-rabbit AlexaFluor 647 (goat)	ThermoFisher Scientific	CAT#A-21244; RRID:AB_2535812

**Chemicals, Peptides, and Recombinant Proteins**		

AlexaFluor-488-conjugated phalloidin	ThermoFisher Scientific	CAT#A-12379; RRID:AB_2315147
DAPI	SIGMA	CAT#D9542
BSA	SIGMA	CAT#A9647
Triton	SIGMA	CAT#X-100
Halocarbon oil 200	Polysciences	CAT# 25073-50
Paraformaldehyde 4%	EMS	CAT#157-4

**Critical Commercial Assays**		

Vectashield	Vector laboratories	CAT#1200-10
24-well cell culture plates	Falcon	CAT#353047

**Experimental Models: Organisms/Strains**		

*D. melanogaster* Oregon-R	BDSC	CAT#5
*D. suzukii* WT3	[23]	N/A
*D. biarmipes* WT	[9]	N/A
*D. melanogaster* 19D09-Gal4	BDSC	CAT#45833
*D. melanogaster* UAS-nlsDsRed	BDSC	CAT#8546, #8547
*E-cad::GFP*^*KIn*^*, sqh–Sqh::mCherry*	[14]	N/A

**Software and Algorithms**		

ImageJ/Fiji	[24]	https://fiji.sc/
Tissue Analyzer (Fijiplugin)	[25]	https://grr.gred-clermont.fr/labmirouse/software/WebPA

**Other**		

Quantitative data used to build the different figure panels	This paper	Data S1

### Contact for Reagent and Resource Sharing

Further information and requests for resources, raw images, movies and reagents should be directed to and will be fulfilled by the Lead Contact Benjamin Prud’homme (benjamin.prudhomme@univ-amu.fr).

### Experimental Model and Subject Details

#### Sex

Females were used for all experiments.

#### Developmental stage

Larval stages from 50% of larval development onward were used for larval experiments. Pupae aged for 18, 24, 30, 36, 48, 54 hours after puparium formation at 25°C were used for pupal staining.

#### Health/immune status

All animals used in this study were healthy.

#### Whether subjects were involved in previous procedures

The animals used in this study were not involved in previous procedures

#### Whether subject is drug or test naive

N/A

#### Genotypes of experimental models

For the quantification of cell number and size across entire ovipositor plates in pupal development, we used the *D. melanogaster* E-cadherin::GFP knock-in line [14]. For the characterization of the larval ovipositor primordium the lines 19D09-Gal4 (BDSC #45833) and UAS-nlsDsRed (BDSC #8546, #8547) were used. For live imaging of the ovipositor in *D. melanogaster* we used the E-cadherin::GFP knock-in line *E-cad::GFP*^*KIn*^*, sqh–Sqh::mCherry* [14]

#### Species/strains of experimental models

For *D. suzukii*, we used the genomic line WT3 for all experiments [23]. For *D. melanogaster*, unless otherwise noted, Oregon-R was used as the wild-type stock.

#### Husbandry and housing conditions of experimental animals

All *Drosophila* stocks were raised and all experiments were performed on homemade Nutri-Fly medium (https://bdsc.indiana.edu/Fly_Work/media-recipes/germanfood.htm). For *D. suzukii*, a strip of Whatmann filter paper was added to the culture vials to facilitate pupation.

In order to generate the full range of viable adult body size in *D. melanogaster* and *D. suzukii* we manipulated the diet. In outline, three cohorts of eggs are laid, 24 hours apart in age. At the appearance of the first wandering larvae and pupae in the oldest cohort, starvation is applied to all three cohorts simultaneously. Individual larvae are removed from each cohort, separated into empty plastic culture vials (with moistened foam plugs) and left at 25°C to pupate.

### Method Details

#### Allometry of ovipositor length and body size

We used ventral pupal area to measure overall body size [26, 27]. Individual pupae are positioned ventral side up on a glass slide and photographed on a Leica Z6 Apo microscope using a ProgRes C5 camera (Jenoptik). Pupal images are thresholded and segmented, and the resultant ventral pupal area measured, using ImageJ/Fiji [24]. Pupae are returned to the vials and left at 25°C until eclosion. Eclosed flies are stored in ethanol at −20°C until dissection. Ovipositors are dissected from the abdomen, and mounted underneath a coverslip in homemade Hoyer’s medium (15 g of gum arabic is dissolved overnight at 60°C in 25 mL of H_2_O in a glass beaker with a magnetic stirrer. 100 g of chloral hydrate is added to the solution. After the chloral hydrate has dissolved, 10 g of glycerol is added. The solution is filtered after a 30 min centrifugation at 10,000 g). Images were taken on an Axio Imager.M2 (Zeiss) microscope using an AxioCam HRc camera. Length and width of the ovipositor plates were measured in ImageJ/Fiji [24]. The length is measured as the distance from the proximal-most point (where the plate meets a bridge of cuticle that connects the two plates) to the base of the distal-most bristle. Using the perpendicular bisector of the length, we measure the width as the distance from the midpoint to the inner margin of the ovipositor plate (see Figures 1B and 1C). Using the *smatr* package in R, the line of best fit was estimated using standardized major axis regression, the data were plotted and the slopes of the regressions for *D. melanogaster* and *D. suzukii* were estimated and compared with the common slope test [28, 29].

#### Growth trajectory of the genital discs

In order to collect synchronized samples of female genital discs at different time points in the third larval instar, *D. melanogaster* and *D. suzukii* wild-type stocks were transferred to new food vials and allowed to lay eggs for 4 hours at 25°C. These 0-4 hour egg cohorts were then allowed to develop at 25°C until the desired time point. For *D. melanogaster*, we collected larvae at 72, 96 and 120 hours after egg laying (h AEL). For *D. suzukii*, due to the extended larval period, we collected larvae at 72, 96 and 120 and 144 h AEL. For the 72 h AEL time class, we exclusively selected L3 larvae. L2 and L3 larvae can be distinguished by the morphology of the anterior spiracles, which are clubbed and branched, respectively. The larvae were sexed using the size of the gonad, which can be recognized in lateral profile as a sphere of translucent tissue against the white background of the adipose tissue. Female larvae have distinctly smaller gonads. Also, to minimize the variability within each time class, we deliberately selected by eye for larvae at the larger end of the size range. In essence, we were selecting for larvae that had been laid earlier in the permitted window, or grown faster since egg laying. This meant that we were comparing a more consistent group of larvae between time classes and between species than if we had applied no size selection. Genital discs were dissected, stained for DAPI and E-cadherin, imaged and processed as described below. To measure overall disc area, the outline of each genital disc was manually delimited, stored as an ROI and measured in ImageJ/Fiji [24]. For each disc, the apical area of a sample of ventral cells was also measured. Confocal stacks of the E-cadherin staining were acquired and individual slices from the ventral part of the disc were selected, segmented and cell apical area measured using the Tissue Analyzer plugin in ImageJ/Fiji [24, 25]. From this, we estimated ventral cell density as the total number of cells in the sample divided by total area occupied by these cells. On the assumption that ventral cell size is relatively homogeneous, we then estimated cell number by multiplying cell density by overall disc area. Plots of overall disc area, cell apical area and cell number against absolute or relative developmental time were generated in Microsoft Excel. Relative developmental time was calculated by expressing each time point (h AEL) as a percentage of the time at which the first puparia appeared (i.e., the onset of pupation, which was 120 h AEL and 144 h AEL for *D. melanogaster* and *D. suzukii*, respectively).

#### Immunohistochemistry

Larval genital disc were dissected in PBS and fixed in 4% paraformaldehyde for 25 min at room temperature. After washes in PBS, they blocked with 1% BSA and 0.3% Triton, as in [30]. Primary antibodies used were: guinea pig anti-Teashirt (gift of Roumen Voutev, Richard Mann; 1:1000); rabbit anti-RFP (Rockland; 1:1000); mouse anti-abdominal-A (Ubx/ABD-A FP6.87 DSHB; 1:5); and rat anti-E-cadherin (DCAD2 DSHB; 1:10). All secondary antibodies were used at 1:500: donkey anti-guinea pig AlexaFluor 488 (Jackson ImmunoResearch); goat anti-rabbit AlexaFluor 568 (Invitrogen); donkey anti-mouse AlexaFluor 647 (Invitrogen); and goat anti-rat AlexaFluor 488 (Invitrogen). All discs were counter-stained with DAPI to reveal nuclei and mounted underneath a coverslip with spacers in Vectashield mounting medium (Vector Laboratories). Images were taken on a LSM 510 Meta (Zeiss) confocal microscope.

The start of pupal development was defined by pupation, and developmental time was scored as hours after puparium formation (h APF). Immobile, white pupae were collected from food vials and kept on moist filter paper at 25°C until the desired time point. In order to improve the synchrony of our samples, the color of the pupal case was checked 20 min after collection: all pupae that were still white were retained, but pupae that had turned brown in that time were discarded. The dissection protocol is modified from a protocol generously provided by V. Courtier-Orgogozo. A single pupa is pinned behind the head on a dissection pad, and the anterior and posterior fragments of the pupal case removed using fine forceps. A wide, circumferential incision is made in the abdomen at about two thirds pupal length (from the head). Through this incision, other non-genital tissues and fatty tissue are washed out hydrostatically using a pipette. The pupal genital organ is thus isolated inside a posterior cap of abdominal tissue. Finally, the posterior cap is separated from the rest of the pupa. These posterior caps are easily handled and the intact genitalia can be left inside for staining. The samples can be stored and stained inside custom-made baskets that fit inside the individual wells of 24-well cell culture plates (Falcon #353047). Between 4 and 6 genital samples are collected on ice (a duration of around 30 min), and then fixed for 30 min in 4% formaldehyde in PBS (phosphate-buffered saline). The only exception is for staining with anti-β-catenin in *D. suzukii* that require a heat fixation method (originally developed for use in *Drosophila* embryos [31]). In outline, first, a solution of 0.4% NaCl/0.03% Triton (abbreviated to “ST”) is brought to the boil in a microwave. Second, using a custom-made basket, the genital samples are immersed in the boiling ST solution for 5 s. Then the samples are immediately transferred to an ice-cold ST solution for ∼60 s, and finally returned to a PBS solution at room temperature. All subsequent steps are carried out at room temperature unless otherwise noted. Fixative is washed out through 3 5-min washes in PBS. Samples are then incubated in blocking buffer (1% BSA, 0.3% Triton in PBS) for 2 hours, and subsequently incubated with primary antibody at the appropriate dilution in blocking buffer overnight at 4°C. Afterward, unbound antibody is removed through 6 × 20-min washes in PBT (0.3% Triton in PBS). Then samples are incubated in secondary antibody in blocking buffer for 2 hours. Finally, samples are washed again in PBT, 5 times for 20 min each. Primary antibodies used were: mouse anti-armadillo (N2 7A1 DSHB; 1:50); mouse anti-Discs large 1 (4F3 DSHB; 1:50); guinea pig anti-Senseless (gift of Hugo Bellen; 1:1000) [13]; rabbit anti-GFP (Invitrogen; 1:500); rabbit anti-RFP (Rockland; 1:1000); and rabbit anti-phospho-Histone H3 Ser10 (Millipore; 1:500). All secondary antibodies were used at 1:500: donkey anti-mouse AlexaFluor 488 or 568 (Invitrogen); donkey anti-guinea pig AlexaFluor 488 (Jackson ImmunoResearch); and goat anti-rabbit AlexaFluor 488, 568 or 647 (Invitrogen). Samples were counter-stained in DAPI to reveal nuclei and mounted underneath a coverslip with spacers in Vectashield mounting medium (Vector Laboratories). For the growth trajectory of the pupal ovipositor and the mitotic index measurements, images of the DAPI, PH3 and Dlg1 staining were taken on a LSM 510 Meta (Zeiss) confocal microscope. For the Ecad::GFP and β-catenin staining of entire ovipositor plates, images were acquired with a 63X objective on a TCS SP8 (Leica) confocal microscope.

#### Measurement of the Teashirt expression domain

We needed to collect synchronized populations of wandering stage, female larvae in both species in order to measure and compare the size of the Teashirt expression domain at the end of larval development. We used a method based on [32]. We prepared a batch of Nutri-Fly food supplemented with a blue dye, so that we could visualize the larval gut contents, and selected wandering larvae with medium blue gut color intensity. The larvae cease feeding and undergo a gut purge during wandering stage, and so this selection improves the synchrony of the larval collections. The larvae were sexed as explained above. The genital discs were dissected, stained and imaged as described above. To quantify the images, the Teashirt expression domain was recognized and delimited by eye, stored as an ROI and its area measured in ImageJ/Fiji [24].

#### Demarcating the pupal ovipositor

Note when we refer to “ovipositor” measurements throughout the text, we are always referring to measurements of the external cell layer of a single ovipositor plate – never the combined area from the pair of plates. In general, using a combination of gene expression and morphological landmarks, the area of the presumptive ovipositor is demarcated manually with the Polygon Selection Tool and measured in ImageJ/Fiji [24]. Between 18 and 30 h APF, the pair of presumptive ovipositor plates is arranged as a pair of lobes on either side of the future egg-laying cavity, lying ventral to the anal plates. We used a set of three consistent landmarks to delimit a triangular area that spans one of these lobes. This area is taken, to a first approximation, as the presumptive ovipositor area. Importantly, the same landmarks are applied to both species, and so any error introduced by the method should at least be of a similar degree in both species, thus permitting a fair comparison. A row of Senseless-expressing cells marks one edge of the lobe (the future ventral margin of the ovipositor plate). We defined the first and second landmark as the dorsal-most and ventral-most Senseless-expressing cell of this row, respectively. The line connecting the first and second landmarks follows the row of Senseless-positive cells. The third landmark is a population of typically 3 Senseless-positive cells found just beyond the dorso-lateral edge of the lobe, in a marginal area that lies between the anal plates and presumptive ovipositor. Straight lines are drawn connecting the first and second landmarks to the third, forming a bounded triangle. By 30 h APF, Senseless expression is often very weak or absent, but in its place, a row of cells with recognizably larger nuclei is visible. These are presumably the polyploid shaft cells of the future bristles, and are thus some of the progeny of the Senseless-positive precursor cells. At 30 h APF, this information from nuclear morphology supplements the weaker Senseless staining in order to place the first and second landmarks at the same dorsal and ventral positions. The third landmark can still be recognized by its position with respect to the presumptive anal plates and ovipositor.

From 36 h APF, the pupal ovipositor is extended as a blade. The ovipositor is demarcated manually using the Polygon Selection Tool in ImageJ/Fiji [24] according to the following method. The dorsal, ventral and distal margins of the blade are easily distinguished from the surrounding background. At the proximal margin, however, the blade connects to the abdominal body wall with no unambiguous morphological feature to define its endpoint. Therefore we developed an *ad hoc* method to define the dorsal and ventral endpoints of the proximal ovipositor, which was then applied in the same way to both species to ensure a consistent and comparable cut-off with the body wall. A dorsal endpoint can be recognized morphologically: the junction of the ovipositor plate and the body wall form a distinctive U-shaped fold, providing a convenient landmark at the vertex of the fold. On the other hand, while the ventral margin of the ovipositor blade can be delimited by its contour, there is no unambiguous feature to determine its endpoint with respect to the body wall. Hence we used a geometric rule to define the ventral endpoint with respect to the dorsal endpoint and the long axis of the ovipositor. With the image oriented such that the long axis of the ovipositor is parallel to the horizontal axis of the image, a straight line is projected ventrally at 90° with respect to the intersection of the dorsal endpoint and the horizontal axis of the image. Then, the intersection of this projected line and the ventral margin of the ovipositor plate defines the ventral endpoint, thus completing a bounded shape that outlines the presumptive ovipositor. With the ovipositor demarcated, in addition to the overall area, the length was measured along the midline running parallel to the PD axis and the width along the orthogonal midline.

#### Quantification of cellular parameters

In order to visualize almost every cell across an entire ovipositor plate, the confocal stacks of the Ecad::GFP and β-catenin staining were projected. The whole-ovipositor projections were generated in ImageJ/Fiji using a custom macro. The projections were segmented and the cell apical area, shape and orientation parameters were measured using the Tissue Analyzer plugin in ImageJ/Fiji [24, 25]. In all cases, some amount of manual correction was required to improve the segmentation. Since each cell was segmented, a raw cell count was produced automatically. However, due to the limits of the projection at the edges of the ovipositor plate and in some internal, folded regions, it was not possible to accurately segment every single cell. Therefore the total segmented area was nearly always smaller than the total measured ovipositor area. We therefore wanted to correct the raw cell counts in order to take account of this additional, un-segmented area. Simply, we estimated the average cell density for each plate using the segmented cells and multiplied this by the missing area (i.e., the difference between the total measured area and the segmented area). We added this correction factor to the raw counts to estimate the total cell number.

To calculate the mean cell number per row and per column for each ovipositor plate, we delineated three rows (running parallel to the PD axis) at dorsal, medial and ventral positions, and similarly three columns (running parallel to the DV axis) at proximal, medial and distal positions (see Figure S2H). We counted the number of cells in each of these rows (or columns) and averaged across the three positions to get an estimate for each plate.

To measure cell shape, an ellipse was fitted to each segmented cell, and the ratio of the long and short axis of the fitted ellipse was calculated. This measure gives an indication of the degree of stretch of the cells.

To measure patterns of cell orientation, we counted the fraction of cells that had their long axis aligned, or otherwise, with respect to the PD axis of the ovipositor. We scored a cell as aligned with the PD axis if the angle created between the cell’s long axis and the ovipositor’s PD axis was less than 45°, and non-aligned if this angle was more than or equal to 45°.

To measure pupal wing apical cell area wings were dissected from 54 h APF pupae (staged as described above) in PBS, and fixed for 30 min at room temperature in 4% formaldehyde in PBS. To visualize actin, wings were stained with AlexaFluor-488-conjugated phalloidin (ThermoFisher Scientific) at a dilution of 1/500 for 2 hours at room temperature, and then washed 3 times for 10 min each in PBS. Wings were mounted underneath a coverslip with spacers in Vectashield mounting medium (Vector Laboratories) and images were taken on a LSM 510 Meta (Zeiss) confocal microscope. Each wing epithelial cell possesses a single hair in the adult. During development, individual cells can be counted easily due to a distinct actin bundle that underlies the forming hairs in each cell. Using a sample of cells in a consistent, anterior distal region of the wing, cell size was estimated in ImageJ by counting the number of cells present in a square of known area [18]. The same protocol was used for both *D. melanogaster* and *D. suzukii*.

All pupal ovipositors were stained with DAPI to visualize the nuclei. For each nucleus, the cross-sectional area was measured at its widest point. The nuclear outlines were delimited manually, stored as ROIs and measured in ImageJ/Fiji [24].

For the cell proliferation analysis, pupal ovipositors at different time points were double stained for Dlg1 and PH3 as described above. First, confocal stacks were acquired with a 40X objective such that the entire ovipositor was within the field of view. All PH3-positive nuclei that fell within the demarcated ovipositor area (see criteria above) were manually counted in ImageJ. Second, for each ovipositor, confocal stacks of the Dlg1 staining were acquired with a 100X objective for a sample of cells. In order to sample a comparable set of cells across specimens, we used the morphologically recognizable bristle cells (or their precursors) as landmarks to select a consistent central region in each plate (located approximately midway between the distal and proximal extremes). Apical slices were selected from these Dlg1 stacks and segmented with the Tissue Analyzer plugin in ImageJ/Fiji in order to measure cell apical area and estimate cell density. On the assumption of sufficiently homogeneous cell density, total cell number was then estimated from the local cell density and total ovipositor area measurements. The mitotic index was calculated as the proportion of PH3-positive cells.

#### Pupariation curves

In order to collect synchronized samples of first instar larvae, first, *D. melanogaster* and *D. suzukii* wild-type stocks were transferred to plates containing Nutri-Fly and allowed to lay eggs for 2 hours. The eggs were left to develop for 24 hours and hatch. 5 replicates of 30 L1 larvae were collected from the plates and transferred to fresh food vials. This process was repeated on 3 independent days to get 15 replicates in total for each species. The number of larvae that had pupated at a given time after egg laying was scored every 3 to 6 hours during the daytime. All incubations were performed at 25°C.

#### Live imaging and image analysis

Pupae were prepared for imaging as in [33]. White pupae were picked and kept at 25°c for 36h APF before dissection. For dissection, pupae were placed laterally on a piece of tape and the posterior pupal case was removed using forceps. The exposed pupal ovipositor was covered with Halocarbon oil 200. Dissected animals were then taped on a coverslip with the ovipositor lateral part facing the coverslip, ready to be imaged. Imaging of fluorescent animals was performed on a Leica SP8, AOBS, equipped with a white laser and a 63x, N.A. 1.4 oil-immersion objective. Live imaging was performed overnight with a z series of 40-50 planes (spaced by 0.8 μm) acquired every seven minutes.

Ovipositor movies were created from maximum intensity projections using ImageJ/Fiji [24]. Images were segmented and cells tracked using the Tissue Analyzer plugin in Fiji in order to extract stable T1 transitions. A T1 transition is defined as a neighbor exchange between exactly four cells (e.g., A,B,C,D) [15]. In any given T1 transition two (e.g., A,B) out of the four cells involved, that were initially in contact with one another, lose this contact while the two other cells (e.g., C,D) that were initially separated by (A,B) become neighbors. In some cases, T1 transitions appear but are reverted at the end of the acquisition, i.e., the 4 cells involved in the T1 transition recover the configuration they started off with. We exclude these ‘oscillating’ T1s from our analysis since they have no net contribution; in contrast, we take into consideration in our analysis all the remaining (‘stable’) T1s.

When a T1 occurs, we define the angle of converging cells by connecting their two centroids; similarly we define the angle of divergence by connecting the centroids of the two cells losing contact. All angles are measured with respect to a line running through the row of visible sensory bristles of the ovipositor. The average angle of convergence and divergence is the average tensor computed as described in [15]: $Q1=\left(1/N_{c}\right)\sum_{\alpha =1}^{N_{c}}Q_{1}^{\alpha }\;\text{and}\;Q2=\left(1/N_{c}\right)\sum_{\alpha =1}^{N_{c}}Q_{2}^{\alpha },$where $Q_{1}^{\alpha }$ and $Q_{2}^{\alpha }$ are the components of the unit length nematic describing the pair of converging cells α, the summation is over all pairs of converging cells analyzed, $N_{c}$. The axis of the average nematic order $\theta_{n}$ used in plots is defined as: $\theta_{n}=\left(1/2\right)\arctan\;2(Q2,Q1).$The same quantification is used for diverging cells.

#### Modeling of the ovipositor length

We idealized the ovipositor as a rectangle, composed of N cells with *n* cells per row and *m* cells per column (N = *nm*). The length (*L*) of the ovipositor is the average number of cells per row (*n*) x the average length of the cell $\left(L_{c}\right)$ $\left(L=n\;L_{c}\right)$. The tissue anisotropy (σ) isn/m. Therefore, n=mσ = *N*σ/*n*. In turn, $n=\sqrt{N\text{σ}}$. Therefore, $L=\sqrt{N\text{σ}}\;L_{c}$ (1). To calculate *Lc*, the average length of the cells along the length of the ovipositor, we took into consideration the shape of the cells and their orientation with respect to the ovipositor long axis. We fitted an ellipse to the cells and measured the average short $\left(l_{1}\right)$ and long $\left(l_{2}\right)$ axis of the ellipse. The shape of the cell (*s*) is described by the ratio of these lengths $\left(s=l_{2}/l_{1}\right)$. The area (*a*) of the ellipse fitting the cell is a proxy for the area of the cell $\left(a=\pi l_{1}l_{2}/4\right)$. In turn, $l_{1}=4a/\pi l_{2}=4a/\pi sl_{1}=2\sqrt{a/\pi s}$ (2). $l_{2}=4a/\pi l_{1}=4as/\pi l_{2}$. So, $l_{2}=2\sqrt{as/\pi }$ (3).

We found that ∼50% of cells have their long axis $\left(l_{2}\right)$ aligned with the long axis of the ovipositor, therefore the average length of the cell $L_{c}$ is $\left(l_{1}/2+l_{2}/2\right)$. Using the Equations (2) and (3), it means that $L_{c}=\sqrt{a/\pi s}+\sqrt{as/\pi }$ (4). Ultimately, using Equation (1), the length of the ovipositor (*L*) can be expressed as a function of the total number of ovipositor cells (*N*), the tissue anisotropy (*σ*), the average area of the cells (*a*) and the average shape of the cells (*s*):$\;L=\sqrt{N\text{σ}}\left(\sqrt{a/\pi s}+\sqrt{as/\pi }\right)$; or $L=\sqrt{N\text{σ}a/\pi }\;\left(\sqrt{s}+\sqrt{1/s}\right)$ (5). With this equation, we calculated the length of the ovipositor using the species-specific values of the different parameters, and compared these length estimates to the actual, measured lengths. Then, we expressed the length of *D. suzukii* ovipositor $\left(L_{suz}\right)$ as a function of the values of the parameters measured in *D. melanogaster* ($\left(N_{mel},{\text{σ}}_{mel},a_{mel},s_{mel}\right)$, affected by the coefficients $\left(k_{N},\;k_{\text{σ}},\;k_{a},k_{s}\right)$, which transform the values of the parameters measured in *D. melanogaster* into the values measured in *D. suzukii*:$\;L_{suz}=\sqrt{k_{N}N_{mel}k_{\text{σ}}{\text{σ}}_{mel}k_{a}a_{mel}/\pi }\;\left(\sqrt{k_{s}s_{mel}}+\sqrt{1/k_{s}s_{mel}}\right)$.

To isolate the contribution of a single cellular parameter to the *D. suzukii* ovipositor length divergence, we set all the coefficients except one to 1 (i.e., no difference between species), and compared the results to the measured ovipositor length. Measures of $N_{mel}$ = 1619, $a_{mel}=11.68\;\mu m$, $\sigma_{mel}=1.53$, $s_{mel}=1.57$, $N_{suz}$ = 1594, $a_{suz}$ = 20.68 μm, $\sigma_{suz}=2.45$, and $s_{suz}=1.57$ at 54 h APF are derived from 34A, 3B, 3J, and S2F, respectively; and $k_{N}=0.9845$, $k_{a}=1.7705$, $k_{\delta }=1.6013,$ and $k_{s}=0.9363$ are calculated from measures shown in Figures 3A, 3B, 3J, and S2F, respectively.

To measure the contribution of one parameter, or a combination of parameters (e.g., cell apical area and tissue anisotropy), we expressed the estimated length using this parameter(s) as a fraction (in %) of the measured length of *D. suzukii* ovipositor (at 54 h APF).

#### Replication

The number of biological replicates for each experiment is indicated in the corresponding figure legends.

#### Strategy for randomization and/or stratification

N/A

#### Blinding at any stage of the study

N/A

#### Sample-size estimation and statistical method of computation

N/A

#### Inclusion and exclusion criteria of any data or subjects

N/A

### Quantification and Statistical Analysis

Statistical analyses were performed with R (using the *smatr* package for the allometry analysis) for the common slope test, or Microsoft Excel for the Student t test and F test to verify the homoscedasticity of the variances.

## Supplemental Information

Document S1. Figures S1–S3

Data S1. Measurements of Ovipositor Parameters during Larval, Pupal, and Adult Stages, Related to Figures 1, 2, 3, S1, and S2; STAR Methods Excel table containing three tabs describing measurements of (A) adult ovipositors; (B) pupal cell parameters; (C) larval ovipositor primordium parameters.

Document S2. Article plus Supplemental Information
