# Supplementary material for: Evolution of Ovipositor Length in Drosophila suzukii Is Driven by Enhanced Cell Size Expansion and Anisotropic Tissue Reorganization
Source: Curr Biol. 2019 Jun 17;29(12):2075–2082.e6. doi: 10.1016/j.cub.2019.05.020 (PMC6584362; doi:10.1016/j.cub.2019.05.020)
Supplement: Document S1. Figures S1–S3 [file mmc1.pdf]

Current Biology, Volume *29*

Supplemental Information

Evolution of Ovipositor Length in *Drosophila suzukii* Is Driven by Enhanced Cell Size Expansion and Anisotropic Tissue Reorganization

Jack E. Green, Matthieu Cavey, Emmanuelle Médina Caturegli, Benoit Aigouy, Nicolas Gompel, and Benjamin Prud'homme

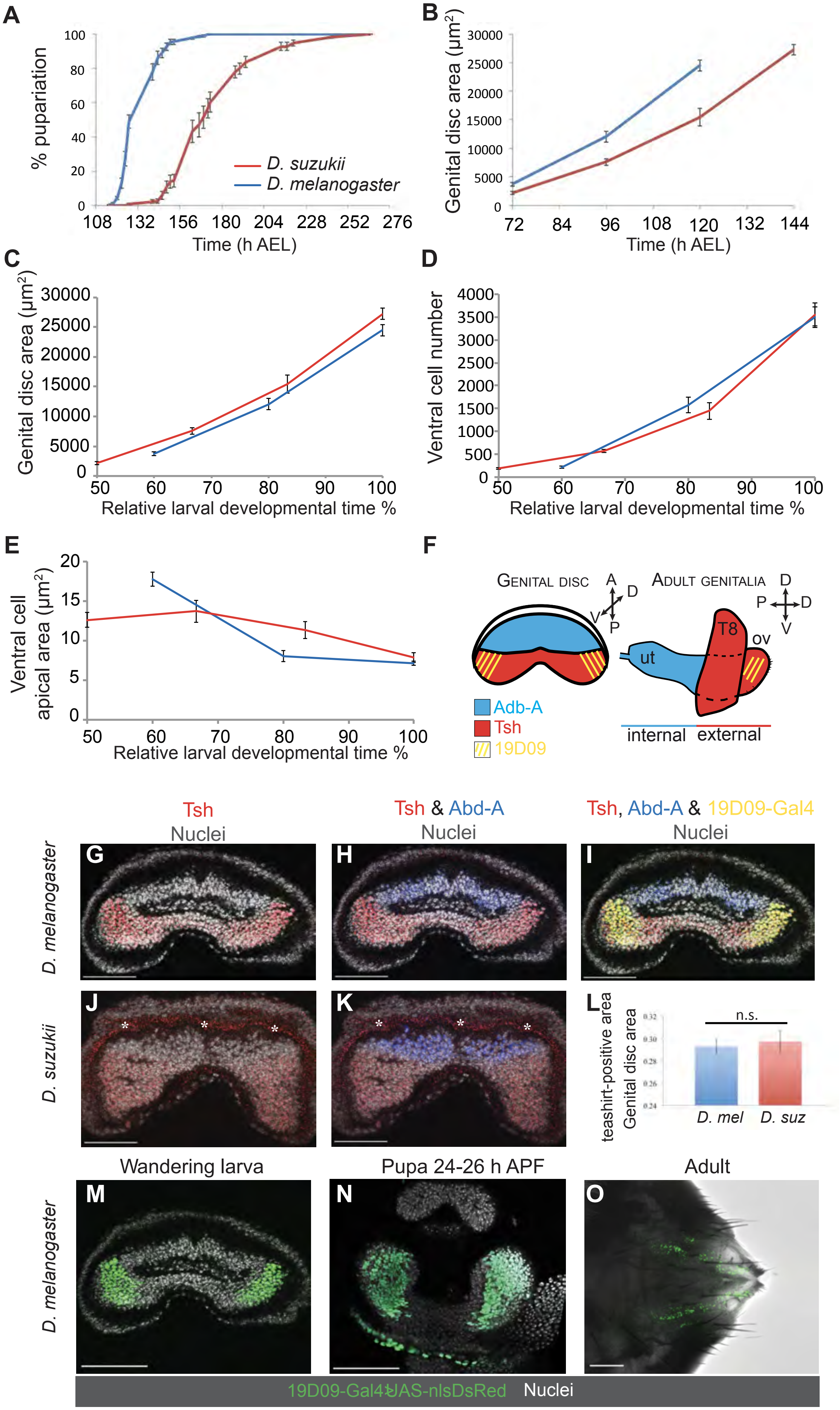

A
% pupariation
100
80
60
40
20
0
108 132 156 180 204 228 252 276
Time (h AEL)
D. suzukii
D. melanogaster
B
Genital disc area (μm²)
30000
25000
20000
15000
10000
5000
0
72 84 96 108 120 132 144
Time (h AEL)
C
Genital disc area (μm²)
30000
25000
20000
15000
10000
5000
0
50 60 70 80 90 100
Relative larval developmental time %
D
Ventral cell number
4000
3500
3000
2500
2000
1500
1000
500
0
50 60 70 80 90 100
Relative larval developmental time %
E
Ventral cell apical area (μm²)
20
15
10
5
0
50 60 70 80 90 100
Relative larval developmental time %
F
Genital disc
A
D
V
P
Adult genitalia
D
P
D
V
T8
ov
ut
Adb-A
Tsh
19D09
internal
external
Tsh
Nuclei
Tsh & Abd-A
Nuclei
Tsh, Abd-A & 19D09-Gal4
Nuclei
D. melanogaster
G
H
I
D. suzukii
J
K
L
n.s.
teashirt-positive area
Genital disc area
D. mel
D. suz
Wandering larva
Pupa 24-26 h APF
Adult
D. melanogaster
M
N
O
19D09-Gal4>UAS-nlsDsRed
Nuclei

Figure S1. The ovipositor primordium does not differ in size by the end of larval development. Related to Figure 2 and Data S1.

(A, B) Larval development is prolonged in *D. suzukii* by approximately one day, and so while the absolute rate of disc growth is slower in *D. suzukii*, the duration of the growth period is extended in compensation. (A) Pupation curve for *D. melanogaster* (blue) and *D. suzukii* (red). h AEL = hours after egg laying. In all graphs, error bars represent the standard error of the mean. The same species colour code applies throughout the figure. (B) Absolute growth in overall genital disc area during third instar larval development.

(C, D) After considering the developmental duration difference, the relative growth trajectories of the female genital disc are essentially indistinguishable during the third larval instar between *D. melanogaster* and *D. suzukii*, for both overall genital disc area and ventral cell number. (C) Growth in the mean, overall genital disc area over relative, developmental time in *D. melanogaster* (blue) and *D. suzukii* (red). (D) Growth in total cell number in the ventral genital disc over time.

(E) Change in mean, ventral cell apical area over time. Although the cell size trajectories differ somewhat between the species, ultimately, we find no significant difference in the apical area of ventral cells by the wandering stage (*D. melanogaster* = 7.13±0.24 μm^2; *D. suzukii* = 7.87±0.64 μm^2; Student's t-test $p > 0.05$). Ventral cell number was estimated using the overall disc area and the apical area of ventral cells that include the primordium of the external genitalia (see Methods). (C-E) In *D. melanogaster*, n=10 discs at 60%, 80%, n=9 at 100%. In *D. suzukii*, n=6 discs at 50%, n=10 at 67%, n=9 at 83%, n=6 at 100%.

(F-L) The relative size of the ovipositor primordium within the genital disc has not changed between species. (F) Schematic representation of the ventral fate map for female, L3 genital discs (left; based on [S1]) and the adult genital structures that the regions give rise to (right; spermathecae and accessory glands are omitted for clarity). The blue, anterior-ventral region expresses *abdominal-A* (Abd-A) and gives rise to the internal genitalia, including uterus (ut) [S2]. We discovered that the expression of the gene *teashirt* (Tsh) is restricted to a population of posterior-ventral cells (in red) that gives rise to the external genitalia, including the eighth tergite (T8) and ovipositor (ov). Tsh has a sharp boundary and a mutually exclusive expression domain with Abd-A (H, K). In addition, Tsh expression overlaps with the expression of a Gal4 line (19D09-Gal4) that marks the ovipositor fate from larval stages to adult in *D. melanogaster* (see M-O). The mutual exclusion of the Tsh and Abd-A expression patterns and the agreement between the Tsh and 19D09-Gal4 spatial distributions support our interpretation that Tsh labels the future external genitalia (F). Tsh expression in the presumptive external genitalia disappears by ~12 h APF (hours after puparium formation). Yellow oblique lines indicate the approximate expression of 19D09-Gal4 in *D. melanogaster*. The compass indicates the orientation: A = anterior; P = posterior; V = ventral; D= dorsal; P = proximal; and D = distal. (G-K) All genital discs are from female, wandering stage larvae, viewed from the ventral side. Scale bar is 50 μm. (G, J) Tsh (red) is expressed in a restricted posterior-ventral cell population, in both *D. melanogaster* and *D. suzukii*, most likely marking the primordium of the external genitalia. (H, K) Tsh has a mutually exclusive expression domain with Abd-A (blue) on the ventral side of the disc in both species. White asterisks in (J, K) indicate non-cellular, non-specific background staining. (I) 19D09-Gal4 (yellow) partially labels the ovipositor primordium in the disc and overlaps with Tsh, but not Abd-A, expression, in *D. melanogaster*. Hence, we can use the Tsh expression domain as a reasonable proxy for the ovipositor primordium. (L) Ratio of Tsh-positive area to overall genital disc

area in *D. melanogaster* (blue; n=16) and *D. suzukii* (red; n=16) female, wandering stage genital discs. n.s. = non-significant, Student's t-test $p > 0.05$. The similarity in the relative area of the Tsh -positive territory between the species at wandering stage strongly suggests that the ovipositor primordia are the same size in *D. suzukii* and *D. melanogaster* by the end of larval development.

(M-0) In *D. melanogaster*, expression of 19D09-Gal4 visualized with UAS-nlsDsRed in (M) genital disc of female, wandering stage larva, (N) pupal ovipositor at 24-26 h APF and (O) adult ovipositor. Scale bar is 50 µm.

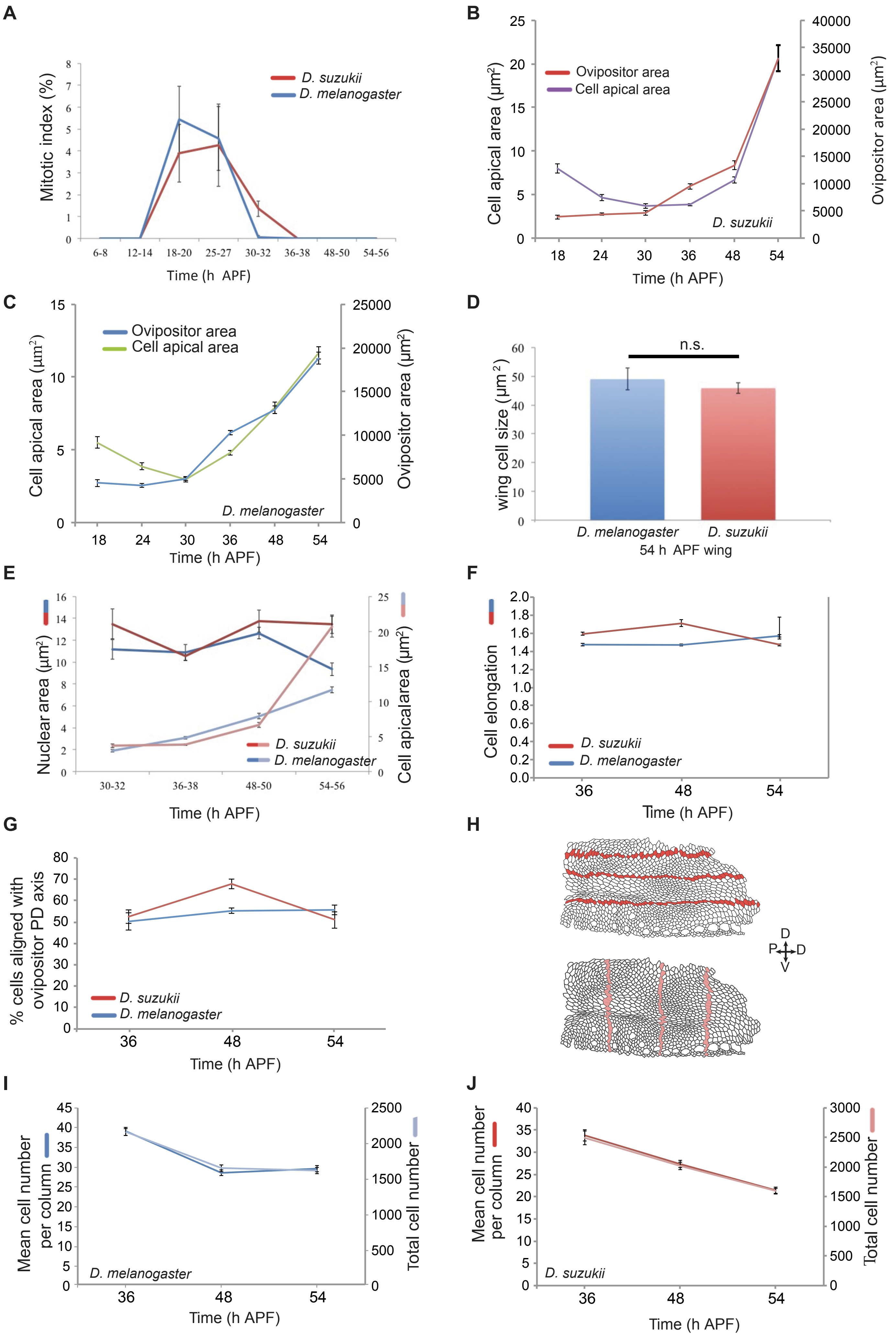

A
Mitotic index (%)
D. suzukii
D. melanogaster
6-8
12-14
18-20
25-27
30-32
36-38
48-50
54-56
Time (h APF)
B
Cell apical area (μm²)
Ovipositor area
Cell apical area
Ovipositor area (μm²)
D. suzukii
Time (h APF)
C
Cell apical area (μm²)
Ovipositor area
Cell apical area
Ovipositor area (μm²)
D. melanogaster
Time (h APF)
D
wing cell size (μm²)
n.s.
D. melanogaster
D. suzukii
54 h APF wing
E
Nuclear area (μm²)
Cell apicalarea (μm²)
D. suzukii
D. melanogaster
30-32
36-38
48-50
54-56
Time (h APF)
F
Cell elongation
D. suzukii
D. melanogaster
Time (h APF)
G
% cells aligned with ovipositor PD axis
D. suzukii
D. melanogaster
Time (h APF)
H
D
P
D
V
I
Mean cell number per column
Total cell number
D. melanogaster
Time (h APF)
J
Mean cell number per column
Total cell number
D. suzukii
Time (h APF)

Figure S2. Comparison of various cellular parameters during ovipositor pupal development. Related to Figure 2, Figure 3, Figure S3 and Data S1.

(A) Mitotic index of the developing ovipositor at 8 time points during metamorphosis, in *D. melanogaster* (blue) and *D. suzukii* (red).

(B, C) Temporal dynamics of cell apical area and ovipositor plate area in *D. suzukii* (B) and *D. melanogaster* (C), showing that the parallel increase in both species

(D) Estimated mean cell size in pupal wing at 54 h APF, in *D. melanogaster* (blue; n=10) and *D. suzukii* (red; n=10). n.s. = non-significant, Student's t-test $p > 0.05$.

(E) Temporal dynamics of nuclear area compared with cell apical area, in *D. melanogaster* and *D. suzukii*.

(F) Temporal dynamics of cell elongation (measured with a cell shape index) in *D. melanogaster* and *D. suzukii*.

(G) Percentage of ovipositor cells with their long axis aligned with the proximo-distal axis of the ovipositor, in *D. melanogaster* and *D. suzukii*.

(H) Drawings showing the outlines of segmented cells for an entire ovipositor plate from a particular *D. suzukii* sample at 48 h APF. Selected rows and columns are highlighted in bold and pale red, respectively, illustrating how the average row and column cell number were estimated for a single plate.

(I, J) The reduction in total cell number in the ovipositor plate correlates in time with the reduction in the number of cell per column of the ovipositor plate, in *D. melanogaster* (H) and *D. suzukii* (I).

h APF = hours after puparium formation. In all graphs, error bars represent the standard error of the mean, and red and blue lines represent data from *D. suzukii* and *D. melanogaster*, respectively.

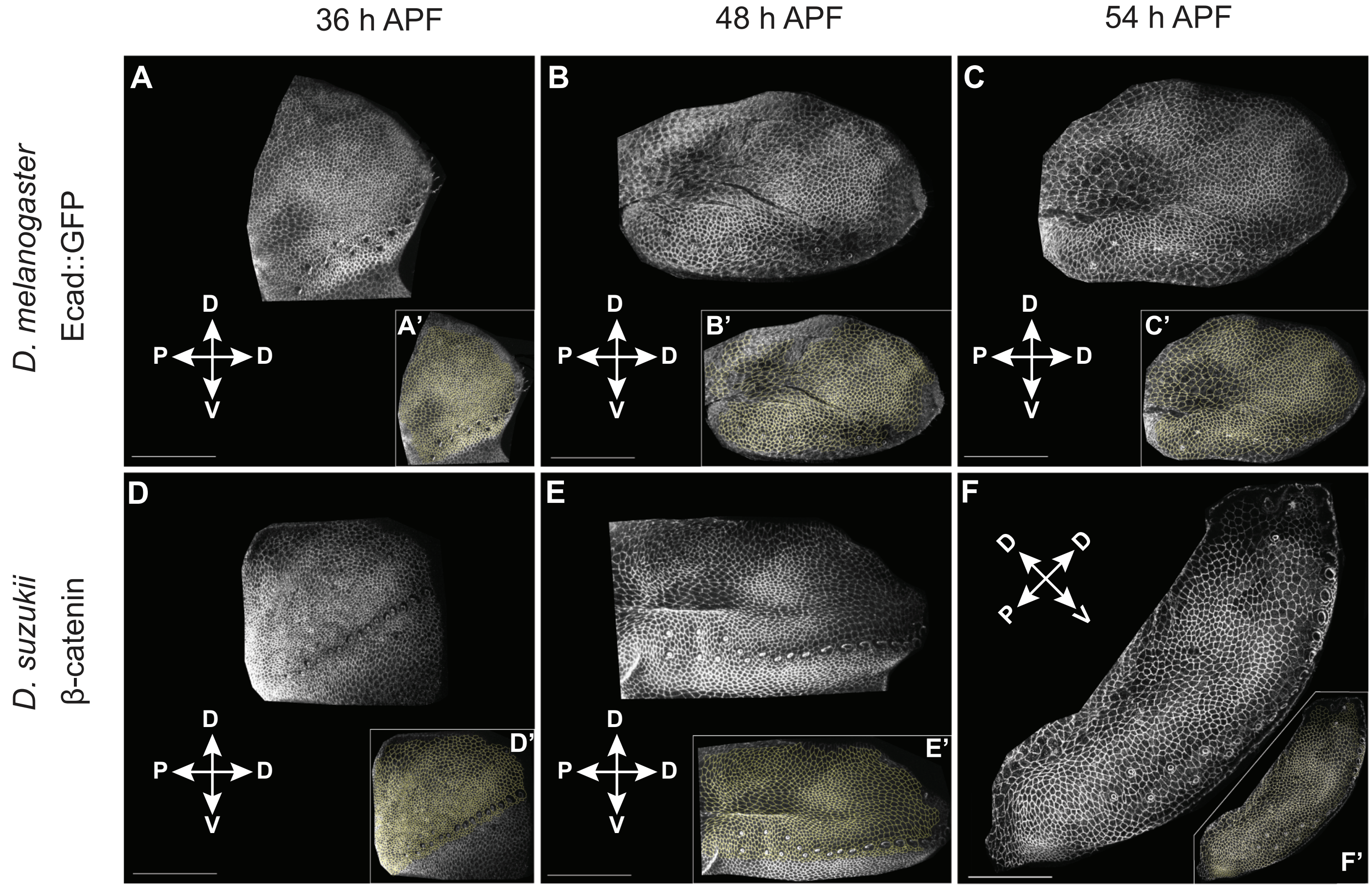
36 h APF
48 h APF
54 h APF
D. melanogaster
Ecad::GFP
D. suzukii
β-catenin
A
A'
B
B'
C
C'
D
D'
E
E'
F
F'
D
P
D
V

Figure S3. Examples showing the segmentation of almost every cell across the entire external layer of one ovipositor plate at various stages. Related to Figure 2, Figure 3 and Figure S2.

(A-C) ECadherin::GFP in *D. melanogaster* at (A) 36 h APF, (B) 48 h APF, (C), 54h APF.
(D-F) ß-Catenin staining in *D. suzukii* at (D) 36 h APF, (E) 48 h APF, (F), 54h APF.
(A'-F') Insets show the overlay with the segmented image (in yellow).

The compass indicates the orientation (P, proximal; D, distal; D, dorsal; V, ventral).
h APF = hours after puparium formation.

Supplemental References

S1. Epper, F. (1983). Three-dimensional fate map of the female genital disc of Drosophila melanogaster. Wilehm Roux Arch Dev Biol *192*, 270–274.
S2. Foronda, D., Estrada, B., de Navas, L., and Sánchez-Herrero, E. (2006). Requirement of Abdominal-A and Abdominal-B in the developing genitalia of Drosophila breaks the posterior downregulation rule. Development *133*, 117–127.
